# Universal Patient Identifier and Interoperability for Detection of Serious Drug Interactions: Retrospective Study

**DOI:** 10.2196/23353

**Published:** 2020-11-20

**Authors:** Howard Michael Sragow, Eileen Bidell, Douglas Mager, Shaun Grannis

**Affiliations:** 1 Express Scripts Inc Franklin Lakes, NJ United States; 2 Express Scripts Inc St. Louis, MO United States; 3 Regenstrief Institute Indiana University Medical School Indianapolis, IN United States

**Keywords:** patient identification, pharmacy benefit manager, interoperability, adverse drug event, identity management, identifier, pharmacy, pharmaceuticals, drug

## Abstract

**Background:**

The United States, unlike other high-income countries, currently has no national unique patient identifier to facilitate health information exchange. Because of security and privacy concerns, Congress, in 1998, prevented the government from promulgating a unique patient identifier. The Health and Human Services funding bill that was enacted in 2019 requires that Health and Human Services report their recommendations on patient identification to Congress. While there are anecdotes of incomplete health care data due to patient misidentification, to date there have been insufficient large-scale analyses measuring improvements to patient care that a unique patient identifier might provide. This lack of measurement has made it difficult for policymakers to balance security and privacy concerns against the value of potential improvements.

**Objective:**

We sought to determine the frequency of serious drug-drug interaction alerts discovered because a pharmacy benefits manager uses a universal patient identifier and estimate undiscovered serious drug-drug interactions because pharmacy benefit managers do not yet fully share patient records.

**Methods:**

We conducted a retrospective study of serious drug-drug interaction alerts provided from September 1, 2016 to August 31, 2019 to retail pharmacies by a national pharmacy benefit manager that uses a unique patient identifier. We compared each alert to the contributing prescription and determined whether the unique patient identifier was necessary in order to identify the crossover alert. We classified each alert’s disposition as override, abandonment, or replacement. Using the crossover alert rate and sample population size, we inferred a rate of missing serious drug-drug interaction alerts for the United States. We performed logistic regression in order to identify factors correlated with crossover and alert outcomes.

**Results:**

Among a population of 49.7 million patients, 242,646 serious drug-drug interaction alerts occurred in 3 years. Of these, 2388 (1.0%) crossed insurance and were discovered because the pharmacy benefit manager used a unique patient identifier. We estimate that up to 10% of serious drug-drug alerts in the United States go undetected by pharmacy benefit managers because of unexchanged information or pharmacy benefit managers that do not use a unique patient identifier. These information gaps may contribute, annually, to up to 6000 patients in the United States receiving a contraindicated medication.

**Conclusions:**

Comprehensive patient identification across disparate data sources can help protect patients from serious drug-drug interactions. To better safeguard patients, providers should (1) adopt a comprehensive patient identification strategy and (2) share patient prescription history to improve clinical decision support.

## Introduction

### Patient Identification in the United States

Interoperability is a key factor in the quality of health care [1-3]. Many anecdotes describe information failing to reach a provider, or providers overlooking records belonging to the same patient, hindering clinical decision making [4,5]. The Centers for Medicare and Medicaid Services Interoperability and Patient Access final rule [6] facilitates better exchange, but without consistent patient identification, its success will be limited. Comprehensive patient identification accurately and efficiently integrates typically fragmented patient data to create a more complete record while mitigating the incorrect linkage of health care data belonging to other patients.

American providers currently do not have a national unique patient identifier to facilitate patient information exchange. Congress, in 1998, prevented the government from promulgating a unique patient identifier by prohibiting funding for such an initiative. Social security number use was not explicitly prohibited, and its use in health care continues. However, privacy concerns persist, and use of social security numbers for health care identity management is steadily declining [7].

The unique patient identifier debate continued until 2019, when the House and Senate bills funding the Department of Health and Human Services diverged. The House bill [8,9] would have enabled the Department of Health and Human Services to promulgate a unique patient identifier; the Senate bill [10] would not. The law enacted in December 2019 was a compromise, requiring that the Department of Health and Human Services report recommendations on patient identification to Congress [11,12]. This study is submitted in part to help inform that recommendation.

While differing models for unique patient identifier assignment exist, a prevailing model in many high-income countries leverages entry events. A central system recognizes an event that occurs once per patient (eg, birth, immigration) and assigns a unique patient identifier. Providers then use the centrally assigned unique patient identifier to identify the patient.

Another model that is common in US health care systems uses demographic matching to assign a unique patient identifier. Providers use demographic information (eg, first name, last name, date of birth, address) to identify the patient, applying the existing unique patient identifier if successfully identified. Otherwise, a new unique patient identifier is assigned. Demographic matching is susceptible to error and as demographics change, providers risk incorrectly duplicating or merging patients.

### Patient Identification and Interoperability in Pharmacy Benefit Managers

Pharmacy is one area of health care where inexact identification can adversely impact patients. Prescription history enables providers to help patients avoid serious drug-drug interactions. Although estimates for serious drug-drug interaction risks vary [13-17], there is ample evidence that they can be dangerous.

To mitigate these risks, both pharmacy benefit managers and dispensing pharmacies perform prospective drug utilization review using prescription history before dispensing drugs [18,19], which assesses the requested medication in the context of the patient’s prescription history and is well-established in pharmacy practice. When electronic review identifies a potentially serious drug-drug interaction, the pharmacy benefit manager alerts the pharmacist through a claim rejection.

Although pharmacy benefit managers process two-thirds of prescriptions in the United States [20], they may lack access to comprehensive prescription histories. During claim adjudication, pharmacy benefit managers aggregate prescriptions filled by multiple pharmacies, creating a history that is more complete than that of any single pharmacy. While pharmacy benefit manager intervention is a secondary defense against serious drug-drug interactions, it augments other medication safeguards.

However, pharmacies can capture prescription history information to which pharmacy benefit managers lack access. Patients may self-pay or obtain reimbursement of prescription costs through manufacturer coupons. Complete visibility into a patient’s prescription history is also limited when a patient transitions between pharmacy benefit managers.

If the dispensing pharmacy system lacks the patient’s prior prescriptions, a labor-intensive process to obtain prescription history may be needed, rendering automated prospective drug utilization review less effective. Consequently, without complete electronic prescription data, automated prospective drug utilization reviews fail to adequately detect potentially serious problems.

Some pharmacy benefit managers have technology to detect records with similar demographics, assigning those to a single unique patient identifier, and using that unique patient identifier during prospective drug utilization review. Other pharmacy benefit managers may use a beneficiary identifier to identify the patient. The latter would treat a record from a new payor as a new patient, omitting the relevant prescription history from prospective drug utilization review and missing serious drug-drug interactions.

Patients risk serious drug-drug interactions going unidentified when their beneficiary identifier changes or when a different pharmacy benefit manager assumes management of their prescriptions. Changes can occur in 1 of 3 ways: (1) The patient changes payor (eg, insurer, employer, labor union, etc) and the new payor uses a different pharmacy benefit manager. Upon a benefit change (employment change, Medicare eligibility, work injury, discount card usage, etc), if the new payor uses a different pharmacy benefit manager, that new pharmacy benefit manager typically does not obtain the patient’s prescription history from the prior pharmacy benefit manager. (2) The payor chooses a new pharmacy benefit manager. When payors select a different pharmacy benefit manager, prescription histories are not always forwarded to the new pharmacy benefit manager. (3) The patient changes payor, but the new and old payors happen to use the same pharmacy benefit manager. The patient adopts a new benefit but keeps the same pharmacy benefit manager.

### Objective

To date, few formal studies have evaluated how interoperability or use of a unique patient identifier affects clinical results. While prior studies have examined potential cost savings [21,22], we are unaware of studies assessing the relationship between use of a unique patient identifier or interoperability and clinical outcomes for large populations.

Regardless of the strategy used to assign a unique patient identifier, increasing evidence links inaccurate identification to poor outcomes [23-28]. Thus, some health care identity experts opine that improved identification methods such as a unique patient identifier may reduce adverse patient outcomes [29]. This study compares the edits resulting from application of a unique patient identifier to prospective drug utilization review with those from not using a unique patient identifier to measure the improvements a unique patient identifier might provide and forecast improvements resulting from broad interoperability.

We hypothesize that using a unique patient identifier to aggregate prescription history for prospective drug utilization review can improve the completeness of patient prescription history, yielding more accurate detection of drug-drug interactions. Our primary objective was to quantify the extent to which a unique patient identifier can improve prospective drug utilization review’s ability to identify serious drug-drug interactions compared to that when using a beneficiary identifier. To contextualize the rate of missing alerts, we measured how often patients migrate between pharmacy benefit managers.

Our second objective was to forecast the improvement in prospective drug utilization review accuracy under the assumption that clinical decision makers have comprehensive access to patient prescription history enabled by a broadly available unique patient identifier.

## Methods

This retrospective analysis uses serious drug-drug interaction alerts that were provided to retail pharmacies at the time of adjudication from September 2016 to August 2019 among a patient population of 49.7 million serviced by a large national pharmacy benefit manager. The pharmacy benefit manager aggregates prescription history data for real-time prospective drug utilization review using proprietary deterministic algorithms to link records to the same unique patient identifier. While no algorithm is perfect, a unique patient identifier can improve patient record completeness [30].

Drug interaction alerts are triggered by a prescription and a precipitating claim. For our first objective, we determined whether each serious drug-drug interaction alert was captured using the same health insurance identifier for both the prescription and precipitating claim. When the precipitating claim originated under different insurance in the absence of a unique patient identifier, we assumed that the pharmacy benefit manager failed to detect serious drug-drug interaction and generated no alert. When the unique patient identifier identified the precipitating claim, enabling prospective drug utilization review to trigger a serious drug-drug interaction alert despite different insurance, we called this event a *crossover alert*. We measured how often crossover alerts occur, relative to all alerts.

For our second objective, we categorized each serious drug-drug interaction alert into 1 of 3 outcomes:

1. Override: The patient receives the medication subsequent to internal pharmacy review within 14 days of the serious drug-drug interaction alert.
2. Replacement: The patient receives another medication treating the same condition within 14 days.
3. Abandonment: The patient did not receive a prescription for another medication treating that condition within 14 days.

We performed chi-square and student *t* tests on bivariate findings and used logistic regression to identify factors correlated with crossover alerts and each of the 3 outcomes (abandonment, override, and replacement). We examine covariates with nonscalar data separated into dichotomous factors representing the more commonly occurring values, including drug, by First Databank specific therapeutic class; Drug Enforcement Agency schedule; month and year of service; type of pharmacy benefit, including Medicare Part D; Exchange Plan under the Affordable Care Act; Medicaid; and Other, including commercial and employer plans; patient age and gender as reported by the payor. We present odds ratios from the logistic regressions alongside the bivariate findings in each of the specific results sections. We also performed a multinomial logistic regression for abandonment and replacement compared to override, in order to rule out inflation of any significance measurements.

Using the serious drug-drug interaction crossover alerts observed and the market share of the pharmacy benefit manager population studied and applying national proportional weights for gender and age distributions, we estimated rates of missing alerts for the entire US insured population.

We assumed that patients randomly remain or transition from their pharmacy benefit manager each year. New health insurance identifiers are typically assigned not by the pharmacy benefit manager but by the payor. Except for patients choosing a new Medicare Part D plan, a patient does not directly select a pharmacy benefit manager, and patients do not choose a new employer on the basis of the pharmacy benefit manager serving the employees. We also assumed that other factors influencing serious drug-drug interactions (ie, demographics, prescribing patterns, self-pay rates, etc) in the observed and unobserved populations were similar.

Crossover alerts were observed in a subset of the US population. We assumed that if pharmacy benefit managers could access prescription records for the remaining population using a common unique patient identifier, crossover alerts of serious drug-drug interactions would reflect all transitioning patients, rather than just those transitioning within a pharmacy benefit manager. Alerts would increase by a proportion that we labeled a *proportionality factor*, which was defined as the US insured population (91.5% of 328 million) [31] divided by the population studied. We again used age and gender weightings to estimate annual serious drug-drug interaction alerts for the entire US population. We estimated an additional crossover alert percentage by multiplying the crossover alert percentage by the proportionality factor.

While serious drug-drug interaction alerts resulting in replacement or abandonment can improve outcomes, we assumed that overridden alerts do not. To estimate annual unidentified alerts that might have helped prevent a contraindicated dispensing, we counted only the proportion that would have resulted in abandonment or replacement as the product of (1) annual national alerts, (2) the crossover alert percentage, and (3) the percentage of serious drug-drug interaction alerts abandoned or replaced.

To contextualize the rate of missing alerts, we measured how often patients migrate between pharmacy benefit managers independently of payor transition. We identified patients taking 2 commonly prescribed medications with indications for long-term preventative therapy for highly prevalent chronic conditions: atorvastatin, indicated for hyperlipidemia, and amlodipine besylate, for hypertension. We expected a high proportion of these patients would have regular claims for these medications throughout the study period. We select patients aged 38 to 48 years old, a range associated with a 2-year mortality rate lower than 1% [32] and identified 2 cohorts: one with at least 2 claims for 1 or both of these medications in 2017, and one with at least 2 claims in the year 2019. For the 2019 cohort, we determined the proportion of patients that were present in 2018 and 2017. For the 2017 cohort, we measured yearly attrition rates in 2018 and 2019.

## Results

### General

For the 49.7 million patients (16.5% of insured population) included in the analysis, 1,436,799,263 total claims were processed during the study period.

From those claims, prospective drug utilization review identified 242,646 serious drug-drug interaction alerts. Among those alerts, 2388 (0.98%) were crossover alerts. Consequently, approximately 1% of all serious drug-drug interaction alerts would not have been detected, were the prospective drug utilization review limited to histories linked to the patient health insurance identifier. Since 16.5% of the insured population had 242,646 serious drug-drug interaction alerts in 3 years, we estimated the US insured population has 458,285 annual serious drug-drug interaction alerts (age- and gender-adjusted).

### Alert Results

Of the 242,646 serious drug-drug interactions, 16.5% (40,128) were abandoned, 73.5% (178,239) were overridden, and 10.0% (24,279) were replaced. Crossover alerts were overridden and abandoned at rates indistinct from those of noncrossover alerts (noncrossover abandoned: 39,601/240,258, 16.5%, crossover abandoned: 527/2388, 22.1%; *P<*.001; noncrossover overridden: 176,551/240,258, 73.5%, crossover overridden: 1688/2388, 70.7%, *P*=.002). Significantly fewer crossover alerts were replaced compared with noncrossover alerts (173/2388, 7.2% vs 24,106/240,258, 10.0%, *P*<.001).

### Month

**Figure 1:**
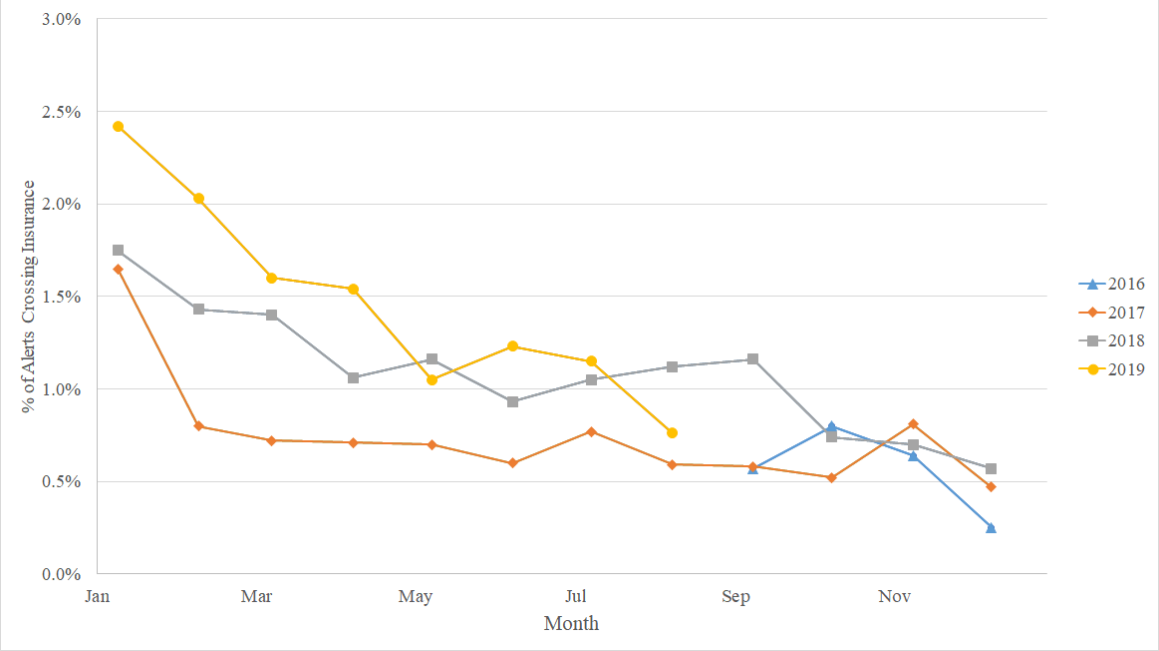
Monthly percentage of alerts crossing insurance identifier (September 1, 2016 to August 31, 2019).

Figure 1 shows crossover alerts occurred significantly more often in January (414/21,801, 1.9%; *P*<.001), February (276/20,226, 1.4%, *P*<.001) and March (262/21,670, 1.2%, *P*<.001), while no differences were noted for the remainder of the year. Multivariate analysis showed that alerts in January were 2.44 (95% CI 2.18-2.74) times more likely to be crossover alerts, those in February were 1.75 (95% CI 1.53-2.00) times more likely to be crossover alerts, and those in March were 1.52 (95% CI 1.33-1.74) times more likely to be crossover (*P*<.001).

### Benefit Type

**Figure 2:**
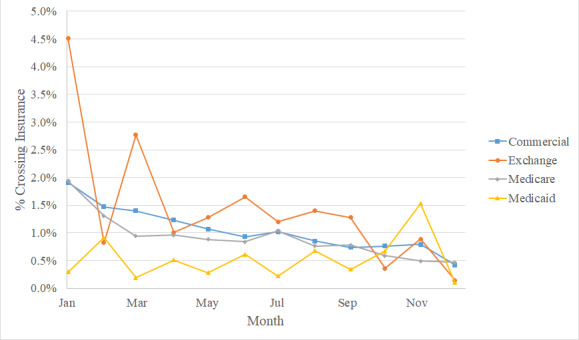
Monthly percentage of alerts crossing insurance identifier by payor type (September 1, 2016 to August 31, 2019).

Figure 2 indicates that among Medicaid beneficiaries, crossover alerts occurred less often (60/11,668, 0.5%; *P*<.001) and did not spike in the first quarter. The percentage of serious drug-drug interaction crossover alerts among patients enrolled in an Affordable Care Act Exchange Plan was higher (85/6173, 1.4%; *P*=.002).

### Age

**Figure 3:**
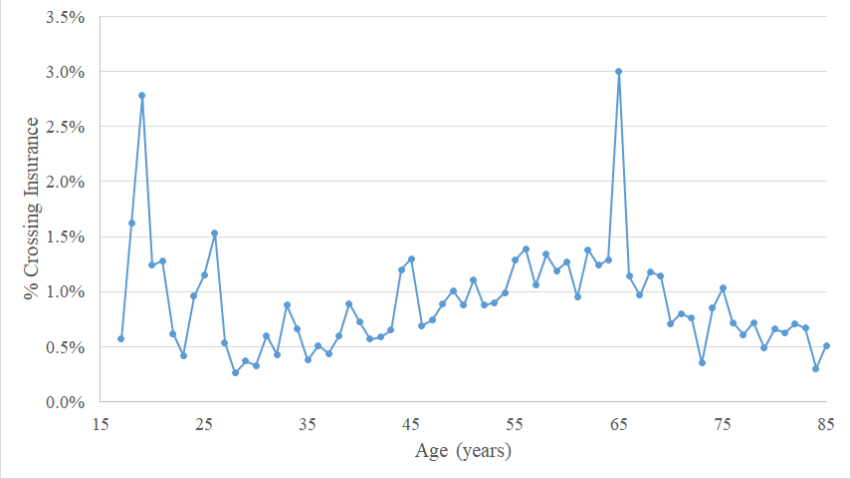
Percentage of alerts crossing insurance identifier by age (September 1, 2016 to August 31, 2019).

Figure 3 highlights that crossover alerts occurred more often at age 19 (7/252, 2.8%; *P*=.004), 5.37 times more likely than patients at ages other than 19, 26, and 65 (95% CI 2.50-11.47; *P*<.001), and significantly more often at age 65 (193/6431, 3.0%; *P*<.001), 2.95 times more likely (95% CI 2.53-3.45; *P*<.001). The rate of crossover alerts was directionally higher in bivariate analysis (12/783, 1.5%) at age 26, but not at the level of statistical significance (*P*=.12). However, multivariate results found that patients aged 26 years were 2.38 times more likely to have crossover alerts (95% CI 1.26-4.47; *P*=.007). Patients younger than 17 years of age seldom experienced serious drug-drug interaction alerts (720/242,646 or 0.3% of the total alerts).

### Patient Movement Between Pharmacy Benefit Managers

We found 373,929 patients between 38 and 48 years of age with at least 2 claims for atorvastatin or amlodipine besylate during 2017. Of those, 69,500 (18.6%) had no claims processed by this pharmacy benefit manager during 2018, and 117,069 (31.3%) had no claims processed by this pharmacy benefit manager during 2019. We similarly found 412,101 patients who had at least 2 claims for atorvastatin or amlodipine besylate during 2019, when they were between 38 and 48 years of age. Of those, 76,222 (18.5%) had no claims processed by this pharmacy benefit manager in 2018, and 147,520 (35.8%) had no claims processed by this pharmacy benefit manager in 2017. These findings confirm our assumption that patients move regularly between pharmacy benefit managers.

### Therapeutic Class

Antibiotics generated the most serious drug-drug interaction alerts overall. Macrolide antibiotics represented 22.5% (54,667/242,646) of all serious drug-drug interaction alerts, and quinolone antibiotics represented 14.5% (35,083/242,646). The second most common was opioids: opioids with nonsalicylates (eg, acetaminophen with codeine) represented 9.6% (23,323/242,646) of all serious drug-drug interaction alerts, and opioid analgesics (eg, tramadol) represented 7.9% (19,239/242,646) of all serious drug-drug interaction alerts. Third was products treating erectile dysfunction (representing 9.9%, 24,090/242,646) of all serious drug-drug interaction alerts and pulmonary arterial hypertension (representing 3.1%, 7632/242,646) of all serious drug-drug interaction alerts. Both erectile dysfunction and pulmonary arterial hypertension medications contain sildenafil and tadalafil.

The rate of crossover alerts was significantly higher (*P*<.001) among claims for erectile dysfunction medications (487/24,090, 2.0%), pulmonary arterial hypertension (171/7632, 2.2%), and vasodilators (499/20,342, 2.4%) compared to those of all other therapy classes. Multivariate analysis shows that alerts for erectile dysfunction drugs were 3.23 (95% CI 2.70-3.88) times more likely to be crossover, alerts for pulmonary arterial hypertension were 3.48 (95% CI 2.81-4.31) times more likely, and alerts for vasodilators were 3.99 (95% CI 3.35-4.77) times more likely (*P*<.001). Drug classes with lower than average crossover alert rates included macrolide antibiotics 0.8% (430/54,667; *P*<.001), quinolone antibiotics 0.76% (267/35,083; *P*<.001), opioid nonsalicylates 0.5% (126/23,323; *P*<.001), and opioid analgesics 0.5% (89/19,239; *P*<.001).

Using all therapeutic classes as the reference category, the replacement rate was higher among macrolide antibiotics (9237/54,667, 16.9%), which were 1.38 times more likely to be replaced (95% CI 1.34-1.42; *P*<.001); opioid analgesics (3541/19,239, 18.4%), which were 1.92 times more likely to be replaced (95% CI 1.80-2.05; *P*<.001); and opioid nonsalicylates (5521/23,323, 23.6%), which were 2.38 times more likely to be replaced (95% CI 2.22-2.54; *P*<.001). Fewer replacements occurred among erectile dysfunction medications (333/24,090, 1.4%), which were 0.52 times less likely to be replaced (95% CI 0.49-0.55; *P*<.001); vasodilators (233/20,342, 1.1%), which were 0.41 times less likely to be replaced (95% CI 0.38-0.44; *P*<.001); and pulmonary arterial hypertension medications (25/7632, 0.32%), which were 0.39 times less likely to be replaced (95% CI 0.32-0.49; *P*<.001). Abandonment was infrequent for macrolide antibiotics (3615/54,667, 6.6%, *P*<.001) but common for erectile dysfunction (8946/24,090; 37.1%, *P*<.001) and pulmonary arterial hypertension (5888/7632, 77.2%, *P*<.001) medications.

### Gender

Males exhibited a higher proportion of crossover alerts than females (males: 1655/132,449, 1.3%; females: 733/110,197, 0.7%; *P*<.001). However, multivariate analysis indicated results were not significant (*P*=.38)

### Additional Crossover Alerts Forecasted With Complete Unique Patient Identifier and Information Exchange

The proportionality factor was (1 / 0.165) – 1= 5.06. Assuming an effective unique patient identifier and complete sharing of prescription data, crossover alerts would increase by a factor of 5.06, resulting in a crossover alert percentage of 5.0%, compared to the original 0.98% (2388/242,646). This rate would be greatest in January, when crossovers are more common. Assuming effective unique patient identifier and complete sharing of prescription data, additional crossover alerts found during January, with an observed crossover alert rate of 1.9% (414/21,801), would increase to 9.6% using the proportionality factor.

### Total Estimated Annual Serious Drug-Drug Interaction Alerts Undiscovered

Using the projected crossover alert percentage of 5.0%, our results indicate that, annually, 22,730 serious drug-drug interaction alerts are undetected by the pharmacy benefit manager.

### Total Estimated Annual Serious Drug-Drug Interaction Alerts That May Result in a Contraindicated Dispensing

We estimate that of the 22,730 undetected serious drug-drug interaction alerts, 6023 (26.5%) would have been replaced or abandoned had they been detected. We therefore estimate that undetected serious drug-drug interaction alerts may contribute to up to 6023 annual contraindicated dispensings because the pharmacy benefit manager does not alert the pharmacy.

## Discussion

### Principal Findings

A significant minority of patients moves annually within and among pharmacy benefit managers, increasing the risk for undetected serious drug-drug interaction alerts due to lack of interoperability and consistent identification. Our analysis highlights several important factors associated with these transitions. Understanding these factors both highlights the need for improved interoperability and can inform future interoperability improvements to mitigate clinical risk.

Most prescriptions are dispensed for a maximum supply of 90 days, thus precipitating claims more than 3 months old are unlikely to trigger a serious drug-drug interaction alert. Therefore, the peak in crossover alerts observed January through March may be explained by patient transition to a new, nonintegrated payor, often at the beginning of each calendar year, fragmenting prescription history. The new pharmacy benefit manager accumulates new pharmacy claims as February progresses into March. These new claims increasingly trigger their own serious drug-drug interaction alerts, while crossover alerts requiring an integrated prescription history decrease.

In addition to yearly fluctuations, health insurance transitions are heightened at specific ages. Crossover alerts increase at ages 19 and 26, resulting from transitions from a family plan, and at age 65 from transitions into Medicare.

We hypothesize that lower-income Medicaid beneficiaries, compared to those of Medicare Part D and employer plan beneficiaries, experience fewer crossover alerts for several reasons. When patients transition to Medicaid, they often have no immediate prior coverage and no associated prescription history. Patients with permanent disabilities having lifelong Medicaid eligibility are also unlikely to switch plans. Medicaid-eligible patients can apply throughout the year, and we did not observe seasonal variation in crossover alert rates for Medicaid beneficiaries. In contrast, Exchange Plan patients must choose their insurer at year-end, and they experience more crossover alerts, particularly in January.

The observed increase in crossover alerts associated with sildenafil and tadalafil may result from noncoverage. Prescription plans often deny benefits for erectile dysfunction treatment. Consequently, patients often seek alternative coverage for these medications, which results in prescription history recorded under a different beneficiary identifier. Vasodilators, which interact with sildenafil and tadalafil, produce more crossover alerts. Noncoverage of erectile dysfunction may also explain the higher rate of crossover alerts for males (24,018/24,090, 99.7% of claims for erectile dysfunction products and 5938/7632, 77.8% of pulmonary arterial hypertension products are dispensed to reported males).

We found a statistical but not clinically meaningful difference in pharmacy response to crossover versus their response to noncrossover alerts. The pharmacy benefit manager studied does not disclose to pharmacies whether a serious drug-drug interaction alert is a crossover alert. The alerted pharmacist learns that the patient has a potential conflict, not how the conflict was identified.

While we observed differences between the rates of crossover, replacement, override, and abandonment among the pharmacy chains studied, these differences were not meaningful and did not impact our conclusions. Similarly, differences, though small, were noticed in average days’ supply for crossovers but did not impact our conclusions. We hypothesize that the findings of differences in mean days’ supply between replacements and overrides is related to the dispensed drug. Antibiotics, as opposed to opioids and erectile dysfunction drugs, are more often replaced and more often dispensed for an acute treatment period.

Our results suggest that improved identification and medication history exchange could help pharmacy benefit managers identify up to 5.0% additional serious drug-drug interactions, and in January, up to 9.7% additional serious drug-drug interactions.

### Limitations

It is possible that many serious drug-drug interaction alerts identified by pharmacy benefit managers using a unique patient identifier may also be detected by the dispensing pharmacy. We lack data to determine the proportion of serious drug-drug interaction alerts triggered by both the pharmacy benefit manager and the pharmacy, as well as the proportion identified solely by the pharmacy benefit manager. Nevertheless, it is clear that using a unique patient identifier enhances the pharmacy benefit manager’s ability to identify serious drug-drug interaction alerts. That being said, we do not directly link unique patient identifier usage to improved health outcomes.

While a unique patient identifier appears to be helpful, differing deployments of unique patient identifier may have varying benefits or introduce novel problems. A unique patient identifier with false positive matches may lead to false positive serious drug-drug interaction alerts, and errors in transcribing a unique patient identifier may lead to misidentification. Furthermore, this study does not address the influence of e-prescribing, which may improve the prescriber’s awareness of the patient’s prescription history and thereby reduce the risk of unmanaged serious drug-drug interactions.

Many factors contribute to the feasibility of various strategies for improving identity. Chief among them is accuracy of the matching process, and the corresponding improvement in clinical outcomes. While this study evaluated the clinical outcomes that could be realized through improved identification, we do not address the issues of privacy and security, which we acknowledge have posed significant barriers to deployment of a national unique patient identifier.

Our results may not be generalizable to other health care contexts. Other providers who receive patient data in different ways will face different challenges. The impact of improved identification on clinical outcomes depends on many factors including workflow, data sources, and data quality. Thus, it is likely that providers in other roles who adopt comprehensive patient identification strategies will achieve different degrees of improvement. However, our results suggest that improved identification can improve outcomes, in this case detection of serious drug-drug interaction alerts. Estimates of impact will require experimental verification and further analysis in additional settings.

### Conclusion

Because the US lacks both a comprehensive identification strategy and ubiquitous health information exchange, our results indicate that up to 6023 contraindicated codispensings may go undetected each year among insured patients. Although progress is being made in US health care systems toward more comprehensive interoperability, fragmented information silos remain the status quo. When patients transition to a new insurer or pharmacy benefit manager, their identity and historical prescription data do not seamlessly follow. Subsequently, pharmacy benefit managers may lack both identifying information and historical data.

A prospective drug utilization review process that does not rely upon a comprehensive patient identity strategy is likely to miss serious drug-drug interaction alerts. A pharmacy benefits manager with a significant market share of the US population that uses only an insurance identifier to aggregate patient records for prospective drug utilization review is likely to miss 1% or more of serious drug-drug interaction alerts, even when using patient information that they already possess but have not linked. The risk of missed serious drug-drug interaction alerts is greater when patients commonly move between benefits: each year in the month of January, and at the ages of 19, 26, and 65.

Additional alerts detected solely through the adoption of a unique patient identifier (ie, without interoperable data sharing) are likely to increase in direct proportion to the size of the population a pharmacy benefit manager system serves. A pharmacy benefit manager that serves more than 15% of the US population and begins using a unique patient identifier in prospective drug utilization review (without any new data transfers) is likely to find an additional 1% serious drug-drug interaction alerts among its patients. A pharmacy benefit manager with smaller market share is likely to identify fewer additional serious drug-drug interaction alerts.

Increased prospective drug utilization review alerting rates beyond those achieved with improved identification can likely be realized with routine information sharing between providers. If all pharmacy benefit managers comprehensively exchanged information for the purpose of prospective drug utilization reviews, it is likely that pharmacy benefit managers would identify 5% more serious drug-drug interaction alerts.

However, serious drug-drug interaction alerts in the future will not necessarily be discovered at the pharmacy counter. As electronic health record systems capture more pharmacy claims data, physician office visits should benefit from more complete patient medication history. This additional data may enable electronic health records to identify more serious drug-drug interaction risks before transmitting a prescription to the pharmacy.

In order to minimize serious drug-drug interactions, we must ensure that comprehensive medication history data is available to prospective drug utilization review. Pharmacy benefit managers that have not implemented a comprehensive patient identity management strategy for prospective drug utilization review should consider doing so. In similar fashion, stakeholders across the health care spectrum should consider implementing comprehensive patient identity management and information exchange strategies to minimize medical errors due to incomplete and missing data.

In order to identify more serious drug-drug interaction alerts, pharmacy benefit managers must ensure that prescribing history is available at times of transition. To do so, pharmacy benefit managers should routinely share new patients’ prior history, regardless of whether transitioning payors request the transfer. Other providers in the health care community should also plan to use interoperability standards in order to obtain relevant records about each patient before providing services.

In the short term, until these measures are achieved, pharmacists should be aware that automated prospective drug utilization review is likely to miss nearly 10% of serious drug-drug interaction alerts in January. They should take particular care during January, ensuring awareness of patients with new coverage using potentially conflicting medications.

While issues of privacy and security remain to be addressed, our data shows that consistent identification can help identify additional serious drug-drug interactions. Given the volume of opportunities to improve patient care, the health care system should choose the most accurate identification strategy possible. We hope that others will conduct similar studies in other areas of the health care ecosystem to forecast benefits from patient identification and patient-record sharing.
